# Mental health (GHQ12; CES-D) and attitudes towards the value of work among inmates of a semi-open prison and the long-term unemployed in Luxembourg

**DOI:** 10.1186/1471-2458-8-214

**Published:** 2008-06-18

**Authors:** Michèle Baumann, Raymond Meyers, Etienne Le Bihan, Claude Houssemand

**Affiliations:** 1 INtegrative research unit on Social and INdividual DEvelopment (INSIDE), University of Luxembourg, Luxembourg; 2 EMACS Research Unit, University of Luxembourg, Luxembourg

## Abstract

**Aim:**

To analyse the relationships between mental health and employment commitment among prisoners and the long-term unemployed (LTU) trying to return to work.

**Method:**

Fifty-two of 62 male inmates of a semi-open prison (Givenich Penitentiary Centre, the only such unit in Luxembourg), and 69 LTU registered at the Luxembourg Employment Administration completed a questionnaire exploring: 1) mental health (measured by means of scales GHQ12 and CES-D); 2) employment commitment; 3) availability of a support network, self-esteem, empowerment; and 4) socio-demographic characteristics.

**Results:**

Compared with LTU, inmates were younger, more had work experience (54.9% *vs *26.1%), and more were educated to only a low level (71.1% *vs *58.0%). The link between employment commitment and mental health in the LTU was the opposite of that seen among the prisoners: the more significant the perceived importance of employment, the worse the mental health (GHQ12 p = 0.003; CES-D p < 0.001) of the LTU; in contrast, among prisoners, the GHQ12 showed that the greater the perceived value of work, the lower the psychic distress (p = 0.012). Greater empowerment was associated with less depression in both populations. The education levels of people who did not reach the end of secondary school, whether inmates or LTU, were negatively linked with their mental equilibrium.

**Conclusion:**

The two groups clearly need professional support. Future research should further investigate the link between different forms of professional help and mental health. Randomized controlled trials could be carried out in both groups, with interventions to improve work commitment for prisoners and to help with getting a job for LTU. For those LTU who value employment but cannot find it, the best help may be psychological support.

## Background

Ensuring equality of access to employment among marginalised vulnerable populations is a priority in Europe[1] as is reducing inequality in their access to health care [2]. Fostering positive attitudes towards working for a living has become a key factor in promoting social and professional integration, but are such attitudes predictors of good mental health?

Two studies looking at this question have been performed in the Grand Duchy of Luxembourg. The first involved inmates of the Givenich Penitentiary Centre (GPC), a semi-open prison where the EQUAL-RESET [3] (Economic and Social Reintegration through Education and Work of Detainees) project was initiated. EQUAL-RESET (2005–2008) is part of the EQUAL [4] Community Program, the goal of which is to make prison terms useful by offering inmates education and training, and developing their personal competence and employability. The aim is to help them obtain the skills and motivation to find and maintain employment, and to adapt to the world of work on their release [5].

Factors associated with recidivism and non-reintegration are well documented in the literature. Among the most important are a lack of assurance or self-confidence, a lack of motivation, poor physical and mental health, substance abuse, lack of qualifications, a low level of social skills, limited access to supportive social networks, and stigmatisation linked to being a former prisoner [6-9].

The literature also includes epidemiological studies indicating that mental illness is the most common cause of death in the prison environment [10] and that the rate of mental disorders is higher than in the overall general population [11]. Many observers [12] believe that certain mentally ill people are over-penalised, and incarcerated rather than being admitted to a hospital facility where they really belong. Others emphasise that prison conditions themselves generate psychological difficulties. Indeed, the environment inside a prison, and the rules that govern life there, can be seriously damaging to mental health. Inmates face a number of losses while serving their sentence. They must simultaneously deal with the loss of resources (lodging, employment), relationships (family, friends), and familiarity with daily life. This affects not only their physical health, but also, crucially, their mental balance. The two mechanisms are not, of course, mutually exclusive.

Finally, looking after mental health has always been an underdeveloped aspect of healthcare provision. In the prison environment this is even more the case – one only has to compare the health services offered to European citizens with those offered to prisoners [10].

The second study that was carried out in Luxembourg involved people registered as long-term unemployed (LTU) at the ADministration of EMployment (ADEM). The goals were to develop a tool that would improve our ability to identify people at high risk of becoming LTU, and to make recommendations to the Ministry of Labour in order to increase the psychological and social support they receive – a measure recommended within the National Action Plan in favour of employment.

The deleterious effect of unemployment on well-being is widely recognised. Being unemployed is described in many studies as resulting in a lack of power over one's own life [13]. Accordingly, finding employment is considered to have the potential to help improve mental health, particularly for people with psychiatric problems [14]. A recent meta-analysis showed that the duration of unemployment affects mental health, even if psychological problems do not lead to behaviour that interferes with a return to work [15]. Lack of psychological well-being does not seem to be an obstacle to searching for and obtaining employment. However, the longer the period of inactivity, the less immediately employable the person becomes. Someone who considers work an important aspect of his or her life (someone with so called '*employment commitment*'), is likely to search more actively for employment and may find it more easily. However, trying to find work and failing increases the risk of a decline in mental health [16].

This raises the vital question of the cumulative role of determining factors in the development of social inequalities with respect to health [17]. It is reasonable to postulate that the effect of an event such as imprisonment or unemployment will be greater and more serious in a group already facing disadvantages in health care and, *vice versa*, that the effect of health problems will be greater when the individual faces social disadvantage. In other words, social circumstances and physical characteristics can predict vulnerability to future difficulties [18]. The financial and psychological instability that accompanies negative life events may reveal or revive latent weaknesses that otherwise do not appear and do not affect health. For example, a psychological fragility that causes no inconvenience when a person's working life is stable may deteriorate to the point of depression; similarly, a physical fragility may worsen to the point of sickness. Unemployment affects health only in conjunction with other aspects (family, medical, social) of that person's history [19].

Given the dearth of literature on attitudes towards work and psychological well-being among people at the margins of the labour market, we decided to look at the psycho-socio-economic characteristics of a group of LTU registered for one year at an employment agency (rather than a random group of the unemployed). The study of this issue within two populations – prison inmates and LTU – seemed promising. Better knowledge of the interaction of the factors relevant to mental health and employment can be expected to help the people responsible for social policies and public health to develop improved tools to promote social integration.

Our objective was to analyse and compare the following among prisoners and LTU trying to return to employment: 1) socio-demographic characteristics; 2) mental health; 3) perceived importance of employment; and 4) availability of a support network, self-esteem, and empowerment.

## Methods

### Samples

Of the 62 prisoners present at the time of the survey (all of whom were men), 52 volunteered to participate. The LTU surveyed were part of a cohort of 384 newly unemployed people (all from Luxembourg), who had been followed since registration. All 127 still unemployed after exactly one year were invited by letter to participate, and 69 (54.3%: 26 women and 43 men) volunteered and were included in the study.

### Ethics

Participants were informed of the aims of each study and told that results would be published anonymously.

### Methods

Both groups completed the same instruments. The prison inmates responded to a questionnaire in French or German (those who asked for help had a researcher read the questions and fill in the form). LTU were given a computerised self-administered questionnaire with a choice of language (French, German or Portuguese). At the beginning of the session, a researcher stayed in the room to give instructions and answer technical questions. No computer knowledge was required, as responses were made using a touch-sensitive screen rather than a keyboard or mouse.

### Measurement instruments

Data were collected on four groups of variables:

#### a) Socio-demographic characteristics

Age, sex, nationality, marital status, dependants, employment situation of the spouse, level of education, work experience, number of hours of further education, current and desired professional status, plus self-estimated state of health (feeling unwell or not).

#### b) Mental health

- *Psychological distress *was measured with the 12-item General Health Questionnaire (GHQ12) [20], a very widely used scale validated in several languages, including French, German and Portuguese (all of which were used in this study). It allows for identification of minor psychiatric and psychological problems as well as a general lack of well-being. Example items: "Have you recently been able to concentrate on whatever you're doing?"; «Have you recently lost much sleep over worry?" The internal consistency of the scale is in the 0.80 to 0.90 range, depending on the study. There were four possible responses to each question (0–1–2–3, as in a Likert scale). The higher the total number of points scored, the worse the mental state.

- *Depression *was evaluated with CES-D, a widely used 20-item scale validated by the Centre for Epidemiological Studies [21]. Example items: "During the last week, I felt hopeful about the future", "During the last week, I thought my life had been a failure". We used the already validated French [22] and Portuguese [23] versions but had to produce a German version translated by a native speaker and back-translated for validation. Four possible responses to each item were given, the higher the score, the worse the depressive state.

#### c) Employment commitment

The importance of employment to the respondent was assessed using a scale of 16 items, each with five possible responses. Example items: "I feel I have a goal in life when I work;" "For me, work is not so important". Our French version was translated into German and Portuguese by native speakers and back-translated for verification. The internal consistency of the scale was evaluated on a random sample of the unemployed (N = 384); Cronbach's alpha (0.82) justified the calculation of a global score – the higher the score, the greater the perceived importance of work [24].

#### d) Associated variables

- *Social support network *was assessed in terms of the number of people available to provide help, using an adapted version of the Social Support Questionnaire by Sarasson (SSQ-6 items) [25]. Instead of listing the initials of people who could possibly provide support, respondents were asked only for the number. Example items: "How many people can you really count on to be dependable when you need help?", "With how many people can you totally be yourself?".

- *Self-esteem *was measured using the Rosenberg Self-Esteem Scale, a 10-item scale validated in many languages, for example English [26], French [27], Portuguese and German [28]. We produced the Portuguese version, which was translated by a native speaker and back-translated for validation. The scale considers the overall perception a person has of his or her own value. Responses are in four areas, and the higher the score, the greater the self-esteem. Example items: "I feel that I have a number of good qualities", "I am able to do things as well as most other people". A recent study using the scale in 53 countries reported internal consistency, as assessed with Cronbach's alpha, mainly in the 0.70 to 0.90 range, with an overall mean of 0.80 [29].

- *Empowerment*, an individual's ability to take control of his or her life and be independent, was measured by means of the *Decision-Making Empowerment Scale*[30]. Five factors (*self-esteem-self-efficacy*, *power-powerlessness*, *community activism and autonomy*, *optimism and control over the future*, *righteous anger*) were identified through factor analysis [30] and used in our study. Items were assessed on a four-point scale; the higher the score, the more empowered the respondent. Example items: "Usually, I feel alone", "People have a right to make their own decisions, even if they are bad ones". The original study showed a high level of internal consistency, with a Cronbach's alpha of 0.86 [30]. The American English version was translated into French, German and Portuguese by native speakers and back-translated for verification.

### Statistical analysis

For each variable, the two groups were compared by means of Chi-square tests and Student's t-tests. We performed power calculations for 5- or 10-point differences on a 0 to 100 scale for the Student's t-tests, and for small and medium effect size (respectively 0.1 and 0.3) for the Chi-square tests [31]. We also estimated the observed power for these tests. All power calculations were done for a type I error of 0.05. The influence on mental health of attitudes related to work was explored by means of separate multiple regressions for the two groups (one for the GHQ12 and one for the CES-D) in which the other variables were taken into consideration. A moderated regression with inmates and LTU as the categorical variable was carried out to compare the slope parameters.

#### Preliminary statistical control for the sex variable

The sample of prisoners comprised only men, whereas 26 of the LTU (37.9%) were women. To detect potential bias in the analysis, we compared all variables in unemployed men and women. No significant differences were observed (results available on request), suggesting that gender is not an issue here, and justifying its absence as a factor in the analysis.

## Results

### Socio-demographic characteristics (Table 1)

**Table 1:** Socio-demographic characteristics of the unemployed and of prison inmates.

		Unemployed (N = 69)	Prison inmates (N = 52)	p^1^	1-β^2^ ES^3 ^= 0.1	1-β ES = 0.3	1-β Obs^4^
		
		%	%				
Age	15–24	31.8	21.6	< 0.001	0.131	0.799	0.961
	25–34	13.6	49.0				
	35–44	24.2	15.7				
	45 or more	30.3	13.7				
Native of Luxembourg	Yes	30.3	40.8	0.331	0.196	0.910	0.227
	No	69.7	59.2				
Dependants	No	51.5	59.6	0.489	0.196	0.910	0.146
	Yes	48.5	40.4				
Situation of the spouse	No spouse	53.8	63.3	0.322	0.152	0.849	0.275
	Working	30.8	18.4				
	Not working	15.4	18.4				
Sex	Female	37.9	0.0				
	Male	62.1	100.0				
Married or co-habiting	Yes	49.3	39.2	0.363	0.196	0.910	0.200
	No	50.7	60.8				
Considers self to be unwell	Yes	21.2	18.0	0.846	0.196	0.910	0.073
	No	78.8	82.0				
Level of education	Lower level	34.5	55.8	0.009	0.152	0.849	0.841
	Average level	36.2	36.5				
	Higher level	29.3	7.7				
No. hours of further education	None	58.0	71.1	0.033	0.152	0.849	0.726
	Less than 400	29.0	8.9				
	400 or more	13.0	20.0				
Experience of working (months)	None	42.0	11.8	< 0.001	0.152	0.849	0.962
	Less than 24	31.9	33.3				
	24 or more	26.1	54.9				
Occupational status sought	Management	17.3	4.9	0.002	0.152	0.849	0.969
	Employee	44.2	19.5				
	Labourer	38.5	75.6				
Driving licence	Yes	67.7	46.2	0.031	0.196	0.910	0.667
	No	32.3	53.8				

^1^p: Significance level of Chi square test; ^2^1-β: Power of the Chi square test for given effect size; ^3^ES: Effect size; ^4^1-β Obs: Observed power of the Chi square test.

The prison inmates were a younger population than the LTU, with over-representation of the 25–34 age-group (49.0% *vs *13.6%, respectively). The majorities of both groups were not natives of Luxembourg, and had no spouse or dependants. Approximately 20% considered themselves to be in poor health. A greater proportion of prisoners had experience of being employed for more than 24 months (54.9% *vs *26.1%). Individuals with lower levels of school education were over-represented in this group (55.8%), whereas LTU were distributed more or less equally among the three levels of education – lower, average and higher. Among the prisoners, 71.1% (*vs *58.0% of LTU) had no further education. Of those who had, 28.9% had received less than 400 hours of training (*vs *42.0% of LTU). Fewer had a driver's licence (46.2% *vs *67.7% of LTU). Three-quarters of prisoners (75.6%) planned to find future employment as labourers, compared with only 38.5% of LTU; 19.5% *vs *44.2%, respectively, were looking for a subordinate role, and 4.9% *vs *17.3% aspired to a management position.

Power calculations showed that the probabilities of getting significant results at the 5% level with our sample sizes were satisfactory for a medium effect size (from 0.799 to 0.91) but would probably fail to detect a small effect (probabilities from 0.131 to 0.196). The observed power for the non-significant tests ranged from 0.073 to 0.275, which means that we cannot state with reasonable certainty that there is no difference between the inmates and the LTU with regard to those socio-demographic variables.

### Mental health, psychological and sociological profiles of inmates and LTU (Table 2)

**Table 2:** Mental health, psychological and sociological profiles of prison inmates and of the unemployed.

	Unemployed (N = 69)		Prison inmates (N = 52)		p^1^	1-β^2^ |Δ|^3 ^= 5	1-β |Δ| = 10	1-β Obs^4^
	
	M^5^	SD^6^	M	SD				
General Health Questionnaire score [0–100]	43.2	[21.4]	47.8	[10.3]	0.154	0.400	0.927	0.349
Depression: Global score [0–100]	32.4	[19.1]	32.2	[17.2]	0.953	0.318	0.844	0.050
- factor: Interpersonal difficulties [0–100]	20.5	[23.8]	22.4	[24.7]	0.669	0.200	0.606	0.071
- factor: Depressive mood [0–100]	29.0	[23.3]	28.9	[19.7]	0.985	0.242	0.711	0.050
- factor: Lack of well-being [0–100]	48.7	[23.9]	39.9	[22.5]	0.043	0.214	0.644	0.533
- factor: Recovery [0–100]	30.0	[19.1]	34.5	[22.8]	0.244	0.252	0.732	0.210
								
Self-esteem [0–100]	65.5	[17.5]	75.6	[16.1]	0.001	0.363	0.896	0.904
								
Social network; availability of social support [0–100]	42.4	[24.3]	50.8	[24.8]	0.068	0.196	0.595	0.455
								
Importance of professional role [0–100]	73.8	[14.9]	78.0	[15.3]	0.130	0.434	0.948	0.329
								
Empowerment [0–100]	62.1	[12.2]	69.8	[8.8]	< 0.001	0.730	0.999	0.977
- factor:: Self-esteem/self-efficacy [0–100]	70.9	[19.1]	82.7	[12.5]	< 0.001	0.401	0.928	0.981
- factor: Power-powerlessness [0–100]	47.9	[13.4]	46.3	[17.8]	0.566	0.410	0.934	0.087
- factor: Community activism and autonomy [0–100]	73.9	[14.7]	83.8	[13.9]	< 0.001	0.473	0.966	0.964
- factor: Optimism and control over future [0–100]	61.6	[16.4]	77.7	[14.9]	< 0.001	0.407	0.932	1.000
- factor: Righteous anger [0–100]	45.0	[12.6]	45.8	[22.0]	0.805	0.345	0.877	0.057

^1^p: Significance level of Student's t-test; ^2^1-β: Power of Student's t-test for given absolute value of the difference; ^3^|Δ|: Absolute value of the difference; ^4^1-β Obs: Observed power of the Student's t-test; ^5^M: Mean; ^6^SD: Standard deviation.

The mental health of prisoners and of LTU was similarly low in terms of psychological distress as measured using the GHQ12, or the CES depression scale, although the dimension "lack of well-being" in the CES-D was significantly higher among the LTU (p = 0.043). Prisoners had, on average, higher self-esteem than did unemployed people (p = 0.001). The same was true of empowerment (p < 0.001), particularly the dimensions concerning self-esteem/self-efficacy (p < 0.001), community activism and autonomy (p < 0.001), and optimism and control over the future (p < 0.001).

Probabilities of results being significant at the 5% level if the absolute value of the true difference was 5 points were lower than 0.5 with the exception of the empowerment scale. These probabilities became much more satisfactory, in most cases, when there was a 10-point difference (ranging from 0.595 to 0.999). As for the socio-demographic variables, we found low power for non-significant tests (from 0.05 to 0.455) suggesting that we cannot conclude that prisoners and LTU differ with regard to those characteristics.

#### Comparisons between our results (GHQ12, CES-D and self-esteem) and the literature

GHQ12 scores in prisoners (mean (M) men 47.8, standard deviation (SD) 10.3) and LTU (men: M 44.2, SD 22.6; women: M 42.6, SD 20.5) revealed greater psychological distress than was observed in a random population of British people (men: M 28.3, SD 12.6; women: M 31.9, SD 14.1) [32]. Similarly, CES-D scores among prisoners (M 32.2, SD 1.2) and LTU (M 32.4, SD 19.1) were higher than in a study performed in a general population in Portugal (M 23.2, SD 16.8) [23].

Comparing the present self-esteem results with those in the literature [29], we found that the unemployed had lower scores (M 65.5, SD 17.5) than did populations in Germany (M 72.4, SD 15.7) and Portugal (M 71.0, S.D15.5). The prisoners' self-esteem was lower (M 75.6, SD 16.1) than that of LTU, but higher than among the general population in France (M 66.2, SD 13.9) and Belgium (M 65.5, SD17.6).

We performed Student's t tests to compare the above with the literature. All differences were highly significant (p < 0.001).

### Relationships between attitudes to work and mental health (Tables 3, 4 and 5)

**Table 3:** Relationships between attitudes to work and mental health among prisoners and the unemployed (separated regressions).

			**General Health Questionnaire [0–100]**					**CES-Depression [0–100]**				
			
			b^1^	L95^2^	U95^3^	SE^4^	p^5^	b	L95	U95	SE	p
**Prison inmates**	Intercept		81.4	46.8	116.0	17.0	< 0.001	63.9	9.3	118.6	26.9	0.023
**- employment commitment**	Perceived importance of work		-0.28	-0.50	-0.07	0.11	0.012	-0.02	-0.36	0.32	0.17	0.923
**- socio-psychological variables**	Empowerment		0.23	-0.17	0.63	0.19	0.244	-0.10	-0.72	0.53	0.31	0.752
	Self-esteem		-0.24	-0.47	-0.02	0.11	0.037	-0.11	-0.47	0.26	0.18	0.557
	Social network; availability of social support		-0.06	-0.22	0.09	0.08	0.426	-0.19	-0.43	0.06	0.12	0.136
**- socio-demographic variables **	Age	15–24	0	-	-	-	0.993	0	-	-	-	0.473
		25–34	-0.50	-9.1	8.1	4.2		3.2	-10.4	16.7	6.7	
		35–44	-1.4	-12.3	9.4	5.3		-7.7	-24.9	9.5	8.4	
		45 or more	0.01	-11.0	11.1	5.4		-3.6	-21.1	13.8	8.6	
	Educational level	Lower level	0	-	-	-	0.147	0	-	-	-	0.022
		Average level	4.4	-2.8	11.6	3.5		-16.4	-27.8	-5.0	5.6	
		Higher level	11.9	-0.84	24.7	6.3		-10.4	-30.5	9.8	9.9	
	Duration of work experience	None	0	-	-	-	0.157	0	-	-	-	0.922
		Less than 24 months	-10.5	-21.6	0.63	5.5		-1.6	-19.2	16.0	8.6	
		24 months or more	-5.3	-15.5	4.8	5.0		0.88	-15.1	16.9	7.9	
	Considers self to be ill	No	0	-	-	-	0.119	0	-	-	-	0.802
		Yes	-6.9	-15.6	1.9	4.3		1.7	-12.1	15.5	6.8	

**Unemployed**	Intercept		63.0	36.7	89.3	13.0	< 0.001	85.5	67.5	103.5	8.9	< 0.001
**- employment commitment**	Perceived importance of work		0.47	0.17	0.78	0.15	0.003	0.40	0.19	0.61	0.10	< 0.001
**- socio-psychological variables **	Empowerment		-0.50	-1.1	0.09	0.29	0.092	-0.82	-1.2	-0.42	0.20	< 0.001
	Self-esteem		-0.47	-0.84	-0.10	0.18	0.013	-0.33	-0.59	-0.08	0.12	0.010
	Social network; availability of social support		0.05	-0.14	0.23	0.09	0.603	-0.07	-0.19	0.06	0.06	0.297
**- socio-demographic variables **	Age	15–24	0	-	-	-	0.355	0	-	-	-	0.093
		25–34	0.47	-11.1	12.0	5.7		-8.8	-16.7	-0.91	3.9	
		35–44	8.0	-3.9	19.9	5.9		-5.4	-13.5	2.8	4.0	
		45 or more	10.3	-1.9	22.5	6.0		-0.32	-8.7	8.0	4.1	
	Level of education	Lower level	0	-	-	-	0.017	0	-	-	-	0.026
		Average level	-10.8	-20.2	-1.4	4.7		-7.8	-14.2	-1.3	3.2	
		Higher level	3.6	-6.9	14.2	5.2		0.75	-6.5	8.0	3.6	
	Duration of work experience	None	0	-	-	-	0.040	0	-	-	-	0.973
		Less than 24 months	8.2	-1.6	18.0	4.9		-0.68	-7.4	6.0	3.3	
		24 months or more	-8.0	-20.0	4.0	6.0		-0.71	-8.9	7.5	4.1	
	Considers self to be ill	No	0	-	-	-	0.005	0	-	-	-	0.455
		Yes	15.7	5.0	26.5	5.3		2.7	-4.6	10.1	3.6	

^1^b: Parameter estimate; ^2^L95: Lower limit of the 95% confidence interval; ^3^U95: Upper limit of the 95% confidence interval; ^4^SE: Standard error; ^5^p: Significance level of the F test.

**Table 4:** Relationships between attitudes to work and mental health among prisoners and the unemployed (moderated regression).

			**General Health Questionnaire [0–100]**					**CES-Depression [0–100]**				
			
			b^1^	L95^2^	U95^3^	SE^4^	p^5^	b	L95	U95	SE	p
**Prison inmates**	Intercept		81.4	37.8	125.1	21.9	< 0.001	63.9	20.6	107.2	21.8	0.004
**- employment commitment**	Perceived importance of work		-0.28	-0.55	-0.01	0.14	0.043	-0.02	-0.29	0.25	0.14	0.905
**- socio-psychological variables **	Empowerment		0.23	-0.27	0.73	0.25	0.361	-0.10	-0.59	0.40	0.25	0.695
	Self-esteem		-0.24	-0.53	0.04	0.15	0.096	-0.11	-0.39	0.18	0.14	0.465
	Social network; availability of social support		-0.06	-0.26	0.14	0.10	0.534	-0.19	-0.38	0.01	0.10	0.063
**- socio-demographic variables **	Age	15–24	0	-	-	-	-	0	-	-	-	-
		25–34	-0.50	-11.4	10.3	5.4	0.927	3.2	-7.6	13.9	5.4	0.560
		35–44	-1.4	-15.2	12.3	6.9	0.836	-7.7	-21.3	5.9	6.8	0.265
		45 or more	0.01	-13.9	14.0	7.0	0.998	-3.6	-17.4	10.2	6.9	0.604
	Educational level	Lower level	0	-	-	-	-	0	-	-	-	-
		Average level	4.4	-4.7	13.5	4.6	0.339	-16.4	-25.4	-7.4	4.5	< 0.001
		Higher level	11.9	-4.2	28.1	8.1	0.145	-10.4	-26.4	5.7	8.0	0.202
	Duration of work experience	None	0	-	-	-	-	0	-	-	-	-
		Less than 24 months	-10.5	-24.5	3.6	7.1	0.141	-1.6	-15.5	12.4	7.0	0.823
		24 months or more	-5.3	-18.1	7.4	6.4	0.407	0.88	-11.8	13.6	6.4	0.890
	Considers self to be ill	No	0	-	-	-	-	0	-	-	-	-
		Yes	-6.9	-17.9	4.2	5.5	0.218	1.7	-9.2	12.7	5.5	0.756

**Unemployed**	Intercept		63.0	40.2	85.8	11.5	< 0.001	85.5	62.9	108.2	11.4	< 0.001
**- employment commitment**	Perceived importance of work		0.47	0.21	0.74	0.13	< 0.001	0.40	0.13	0.66	0.13	0.004
**- socio-psychological variables **	Empowerment		-0.50	-1.0	0.01	0.25	0.054	-0.82	-1.3	-0.32	0.25	0.002
	Self-esteem		-0.47	-0.79	-0.15	0.16	0.004	-0.33	-0.65	-0.02	0.16	0.039
	Social network; availability of social support		0.05	-0.11	0.21	0.08	0.554	-0.07	-0.23	0.09	0.08	0.411
**- socio-demographic variables **	Age	15–24	0	-	-	-	-	0	-	-	-	-
		25–34	0.47	-9.6	10.5	5.0	0.925	-8.8	-18.8	1.1	5.0	0.082
		35–44	8.0	-2.3	18.4	5.2	0.126	-5.4	-15.6	4.9	5.2	0.301
		45 or more	10.3	-0.27	20.9	5.3	0.056	-0.32	-10.8	10.2	5.3	0.952
	Level of education	Lower level	0	-	-	-	-	0	-	-	-	-
		Average level	-10.8	-19.0	-2.6	4.1	0.010	-7.8	-15.9	0.34	4.1	0.060
		Higher level	3.6	-5.5	12.8	4.6	0.432	0.75	-8.4	9.9	4.6	0.870
	Duration of work experience	None	0	-	-	-	-	0	-	-	-	-
		Less than 24 months	8.2	-0.34	16.8	4.3	0.060	-0.68	-9.2	7.8	4.3	0.873
		24 months or more	-8.0	-18.4	2.5	5.2	0.132	-0.71	-11.1	9.7	5.2	0.892
	Considers self to be ill	No	0	-	-	-	-	0	-	-	-	-
		Yes	15.7	6.4	25.1	4.7	0.001	2.7	-6.5	12.0	4.6	0.556

^1^b: Parameter estimate; ^2^L95: Lower limit of the 95% confidence interval; ^3^U95: Upper limit of the 95% confidence interval; ^4^SE: Standard error; ^5^p: Significance level of the t-test.

**Table 5:** Slope parameters: differences between prison inmates and LTU (moderated regression).

	D^1^	L95^2^	U95^3^	SE^4^	p^5^
**General Health Questionnaire [0–100]**					
Perceived importance of work	0.76	0.37	1.14	0.19	< 0.001
Empowerment	-0.73	-1.44	-0.02	0.36	0.045
Self-esteem	-0.23	-0.66	0.21	0.22	0.301
Social network; availability of social support	0.11	-0.14	0.36	0.13	0.393

**CES-Depression [0–100]**					
Perceived importance of work	0.41	0.03	0.79	0.19	0.033
Empowerment	-0.72	-1.43	-0.02	0.35	0.045
Self-esteem	-0.23	-0.66	0.20	0.21	0.291
Social network; availability of social support	0.12	-0.13	0.37	0.13	0.349

^1^D: Difference between Prison Inmates and LTU slope parameters; ^2^L95: Lower limit of the 95% confidence interval; ^3^U95: Upper limit of the 95% confidence interval; ^4^SE: Standard error; ^5^p: Significance level of the t-test.

The results of the two models of multiple regression, with which we attempted to explain differences in the two variables of mental health (GHQ12 psychological distress and CES-depression), explained respectively 59.3% and 77.2% of the variance (*adjusted R-square*) among the unemployed, but only 18.4% and 26.1% among prison inmates. Thus, the models provided stronger explanations for the mental health of the unemployed than for that of prisoners. The model of moderated regression explained 52.5% and 58.4% of the variance of the GHQ12 and of the CES-D scores, respectively.

The link between employment commitment and mental health differed between the two populations: among the LTU, the more significant the perceived importance of employment, the worse a person's mental health (GHQ12 p = 0.003; CES-D p < 0.001). In contrast, among prisoners, the GHQ12 showed that the greater the work commitment, the lower the psychic distress (p = 0.012). The estimated difference between these two parameters was 0.76 [0.37; 1.14] and differed significantly from zero (p < 0.001). We also observed another interesting differential effect on empowerment: the greater the empowerment of LTU, the less likely they were to be depressed (p < 0.001), whereas empowerment did not seem to be associated with depression among inmates (p = 0.752, LTU – inmates difference: -0.72 [-.43; -0.02], p = 0.045). The greater the self-esteem, the better the psychological state of both the unemployed (GHQ12 p = 0.013; CES-D p = 0.01) and prison inmates (GHQ12 p = 0.037). Availability of a social support network had no significant effect in either group.

Among the prisoners, a lower level of education (compared with a higher level) contributed to depression (10.4/100 points for the CES-D score) whereas an average education was associated with a lower depression score (p = 0.022). LTU educated to an average level had better mental health than those educated to a lower or higher level (GHQ12 p = 0.017; CES-D p = 0.026). Having little work experience (GHQ12 p = 0.04) and feeling unwell (GHQ12 p = 0.005) were linked to psychological distress (15.7/100 points more, on average).

## Discussion

Prison inmates and LTU are psychologically vulnerable populations at the margins of the labour market. The GHQ12 data presented here reveal psychological fragility and depressed states among prison inmates. The overall socio-demographic profiles of the two groups differed: the socio-professional level of prisoners was lower than that of the LTU; they were younger, less educated, fewer had a driver's licence, and fewer had received further education, more had over 24 months of work experience and planned to seek employment as a labourer. The principal finding of this study was that although our two samples had similar mental health, work commitment was associated in opposite ways with psychological well-being.

Among the prison inmates, employment commitment predicted well-being. In contrast, greater perceived value of work among the unemployed was linked with worse mental health. In short, the factor which was linked to better mental health of the first group was associated with worse mental health of the second. The more employment commitment a prisoner had, the less he seemed to suffer psychologically. In contrast, the more necessary work was to an unemployed person, the worse his or her mental health. This result is in accord with the conclusions of McKee-Ryan and colleagues 2005 [15] that attributing great importance to one's professional role while being forced to remain unemployed produces a negative effect on well-being and mental health.

The education levels of people who did not reach the end of secondary school, whether prisoners or unemployed, were negatively linked with their mental equilibrium. A British investigation into unemployment showed the differential effects of "acquired advantages and disadvantages", a measure related to social affiliation. This study (part of the British "General Household Survey") found that men who have declared a long-standing illness are more likely to be unemployed and inactive than those who have not [33]. Our study also highlights (among the ill compared to non-ill LTU) a possible relationship between feeling ill and experiencing psychological distress. This observation supports the hypothesis that social health inequality is a cumulative process throughout life, whereby social experiences interact with, and contribute to, individual physiology and pathology [34].

Greater empowerment was associated with less depression. On average, prison inmates were more empowered than were the unemployed. The differentiating factors were community activism and autonomy, optimism and control over the future, and self-esteem/self-efficacy. A study that monitored ambulatory patients showed that those who had a full-time job were more empowered than those who did not [35]. In contrast, other research using the same scale as we did, found no difference among people using mental health services, emphasising the importance of workplace climate and its congruence with individual value systems [36].

Positive attitudes towards prisoners among those helping them have been reported to have an important beneficial impact on the effectiveness of various correctional rehabilitation programs and on successful reintegration. A number of strong correlations have been observed between negative attitudes towards prisoners and more pessimistic and punitive answers to general questions about prisoners, crime and punishment. Whether attitudes toward prisoners can be influenced by educational programs and the dissemination of factual information needs to be investigated [37]. These findings could have important implications, particularly for preventive work in prisons.

The present investigation verifies that strong self-esteem is associated with the best mental health among both prisoners and the unemployed. Self-esteem has been widely studied in the field of unemployment, and is recognised as a better predictor of mental health than is finding a job [38]. One study [39] showed that acts of delinquency improved the self-esteem of young men with low socio-economic status, regardless of their mental state. However, among depressed people, self-esteem was unstable. The authors suggest that it should not be considered predictive of mental health in the population they describe

### Limits of the study

Our results should be interpreted with caution for the following reasons: we surveyed only small samples of volunteers (Luxembourg is one of the smallest countries in Europe). Only those prison inmates available at the times allocated for completing the questionnaires participated, and recruitment to the LTU group was limited to individuals who had been unemployed for exactly 12 months. Furthermore, the survey was cross-sectional and declarative, whereas the responses were subjective and dependent on the time of the interview. Conclusions should be drawn only with caution from comparisons of our samples with random populations of other countries, as many sociological, economic, psychological and cultural, factors may play a role.

Our findings are of a correlative nature only, and no conclusions can be drawn about causative links between attitudes with regard to employment commitment and mental health. Complementary longitudinal studies or randomised controlled trials would produce additional information.

## Conclusion

The types of professional support offered to prisoners and LTU should be tested in line with our preliminary results to better adapt them to the precise mental health needs of both populations. Randomized controlled trials could be carried out, with specific interventions for the two groups: prisoners could be helped to develop positive attitudes toward work and given the opportunity, with support, to find employment in semi-freedom (work release) or at the end of their sentences. With LTU, who value employment highly but cannot find it, the principal intervention to test would be help with getting a job.

The mental health profiles of the two groups in our study suggest that if a quick return to employment is not achieved, it stands to reason that psychological or psychiatric support would help them bear the emotional burden.

## Competing interests

The authors declare that they have no competing interests.

## Authors' contributions

MB: Scientific Director of the Equal-reset Project, participated in conceiving the study and writing the manuscript. RM: participated in conceiving the study and writing the manuscript. ELB: conducted the statistical analysis and participated in writing the manuscript. CH: Scientific Director of the Long-term Unemployment Project, participated in conceiving the study and writing the manuscript. All authors have read and approved the final manuscript.

## Pre-publication history

The pre-publication history for this paper can be accessed here:


